# Phenotypic drug discovery: a case for thymosin alpha-1

**DOI:** 10.3389/fmed.2024.1388959

**Published:** 2024-06-06

**Authors:** Enrico Garaci, Maurizio Paci, Claudia Matteucci, Claudio Costantini, Paolo Puccetti, Luigina Romani

**Affiliations:** ^1^San Raffaele Sulmona, L’Aquila, Italy; ^2^Department of Chemical Sciences and Technologies, University of Rome “Tor Vergata”, Rome, Italy; ^3^Department of Experimental Medicine, University of Rome Tor Vergata, Rome, Italy; ^4^Department of Medicine and Surgery, University of Perugia, Perugia, Italy

**Keywords:** phenotypic drug discovery, network pharmacology, thymosin alpha-1, immune regulation, cancer and infection therapy

## Abstract

Phenotypic drug discovery (PDD) involves screening compounds for their effects on cells, tissues, or whole organisms without necessarily understanding the underlying molecular targets. PDD differs from target-based strategies as it does not require knowledge of a specific drug target or its role in the disease. This approach can lead to the discovery of drugs with unexpected therapeutic effects or applications and allows for the identification of drugs based on their functional effects, rather than through a predefined target-based approach. Ultimately, disease definitions are mostly symptom-based rather than mechanism-based, and the therapeutics should be likewise. In recent years, there has been a renewed interest in PDD due to its potential to address the complexity of human diseases, including the holistic picture of multiple metabolites engaging with multiple targets constituting the central hub of the metabolic host–microbe interactions. Although PDD presents challenges such as hit validation and target deconvolution, significant achievements have been reached in the era of big data. This article explores the experiences of researchers testing the effect of a thymic peptide hormone, thymosin alpha-1, in preclinical and clinical settings and discuss how its therapeutic utility in the precision medicine era can be accommodated within the PDD framework.

## 1 Phenotypic drug discovery

In 1928, at St. Mary’s Hospital in London, Scottish researcher Alexander Fleming was studying bacteria. While he was away on vacation, he forgot to cover a petri dish containing staphylococci on his lab bench. When he returned, he found mold had grown on the dish, surrounding the bacteria. He noted the bacteria seemed suppressed and not spreading where the mold was. Fleming identified the mold as *Penicillium* and hypothesized it was producing something that inhibited bacterial growth, which he named “penicillin.” Even though Fleming saw the potential, he did not pursue it further. It wasn’t until the early 1940s that researchers Howard Florey and Ernst Chain at the University of Oxford revisited Fleming’s work. They saw the therapeutic potential of penicillin and worked to isolate and purify it. After extensive efforts, they successfully produced enough penicillin for clinical trials. Penicillin was highly effective in treating bacterial infections, especially against deadly pathogens like *Streptococcus* and *Staphylococcus*. This accidental discovery and the subsequent efforts by Florey and Chain had a transformative impact on the field of medicine, exemplifying a fortuitous observation that turns out to be highly valuable when the researcher is not even looking for it (1).

The above is thus a prototypic example of “serendipitous drug discovery” (2). Whereas serendipitous discovery is characterized by unexpected and unplanned findings that lead to the identification of a drug candidate, phenotypic drug discovery (PDD) refers to an approach in drug development that focuses on the observable characteristics or phenotypes of diseases and their responses to potential drug compounds (3, 4). Instead of targeting specific molecular targets or pathways, PDD aims to identify drug candidates based on their ability to produce a desired effect on the disease phenotype. Thus, PDD and serendipitous discovery are two distinct approaches in the field of drug development, and they differ in their underlying principles and processes. If, on the one hand, serendipitous discoveries occurred when researchers observed unexpected effects of compounds that were initially being investigated for different purposes, PDD, on the other hand, involves screening compounds based on their ability to produce desired phenotypic changes in disease models or patient samples (5, 6). It focuses on observing and understanding the observable characteristics of diseases and their response to potential drug compounds. PDD is particularly useful when the underlying molecular mechanisms of a disease are not fully understood, or when targeting a specific molecular target has not yielded successful drug candidates (6–8). As a matter of fact, PDD allows researchers to explore the complex interactions within biological systems and discover drugs that act through novel mechanisms. Moreover, this approach can lead to the discovery of drugs with greater efficacy and broader therapeutic applications as it takes into account the complex biology of diseases and their interactions with the surrounding environment. Despite these advantages, PDD presents challenges such as hit validation, target deconvolution and safety issues due to the engagement of multiple targets. Box 1 further exemplifies the above concepts pointing to a degree of overlap between PDD and drug repurposing.

BOX 1 **Serendipitous drug discovery, phenotypic drug discovery, and drug repurposing**

1. **Serendipitous drug discovery**: Serendipity refers to the accidental discovery of something valuable while searching for something else. Serendipitous drug discovery occurs when a drug is discovered by chance, without a specific intention or target in mind. Here's an example: Viagra (Sildenafil)—Initially developed as a treatment for angina, researchers discovered during clinical trials that it had an unexpected effect on male sexual function, leading to its use as an erectile dysfunction treatment.

2. **Phenotypic drug discovery**: Phenotypic drug discovery involves screening chemical compounds for their ability to produce a desired therapeutic effect on a biological system, without initially knowing the underlying molecular target. This approach focuses on observing the phenotypic response of cells, tissues, or organisms to identify potential drug candidates. Aspirin (acetylsalicylic acid)—Originally derived from willow bark, aspirin was found to demonstrate antipyretic (fever-reducing), analgesic (pain-relieving), and anti-inflammatory effects, leading to its development and use as a widely used medication.

3. **Drug repurposing**: Also known as drug repositioning or drug reprofiling, drug repurposing involves finding new therapeutic applications for existing drugs that were initially developed for a different indication. Instead of developing a new drug from scratch, researchers explore existing drugs for their potential in treating other diseases or conditions. Thalidomide—initially developed and marketed as a sedative—was later discovered to effectively treat leprosy and multiple myeloma, illustrating its repurposing for different therapeutic purposes.

Indeed, PPD and drug repurposing share such an overlap in the sense that both approaches prioritize the observed effects of a compound on cells, tissues, or organisms, rather than solely focusing on its specific molecular target. The overlap is rooted in the fact that both methods look at the broader, observable effects of a compound on biological systems and can lead to the discovery of new therapeutic applications for existing compounds or the identification of new compounds with therapeutic potential. This can be particularly valuable in situations where traditional target-based approaches have been unsuccessful or when there is a need to find new treatments more efficiently. In some cases, the phenotypic response observed during PDD can lead to the repurposing of existing drugs or identification of potential targets for further drug development. As the overlap between these two approaches arises from the fact that both seek to identify new therapeutic opportunities by leveraging existing compounds, there occur instances whereby compounds identified through phenotype-based screens may have known pharmacological activities that make them suitable candidates for repurposing in other disease contexts. Similarly, drugs identified through repurposing efforts may have broad-spectrum activities that make them suitable for phenotype-based screens in multiple disease models. Box 2 epitomizes the principles that qualify a drug as the result of PDD.

BOX 2 **Identifying novel drugs according to phenotype**

1. **Mode of action discovery**: In phenotypic drug discovery, the drug's mode of action is often identified after the initial observation of the desired phenotypic effect. This is in contrast to target-based drug discovery, where the drug's target is known before its effects are observed. For example, thalidomide, originally designed as a sedative, was later found to have anti-inflammatory properties and is now used to treat multiple myeloma.

2. **Multiple targets or pathways**: Phenotypic drugs often interact with multiple targets or pathways simultaneously, leading to a complex mechanism of action. Examples are the inhibitor of multiple tyrosine kinases, imatinib, for its clinical use for gastrointestinal stromal tumors and metformin, a widely used diabetes drug, affects various cellular processes involved in glucose homeostasis.

3. **Broad-spectrum activity**: Phenotypic drugs sometimes exhibit activity against multiple disease indications or different strains of organisms. For example, the antibiotic penicillin has a broad spectrum of activity against different bacteria and is effective in treating various infections.

4. **Limited target bias**: Phenotypic drug discovery may allow for the identification of drugs that act on targets or pathways not traditionally associated with a particular disease. This can potentially uncover novel therapeutic approaches. One example is sildenafil, initially developed as a drug for hypertension, which was later discovered to be effective for erectile dysfunction.

5. **Complexity of effects**: Phenotypic drugs often have a diverse range of physiological effects beyond the desired therapeutic effect. This can lead to unexpected outcomes, both positive and negative. For example, while the antipsychotic drug thioridazine effectively treats psychosis, it also prolongs the QT interval in the heart, potentially causing cardiac arrhythmias.

Ultimately, the overlap between phenotype-based drug discovery and drug repurposing reflects the potential for cross-fertilization between different drug discovery strategies, leading to the identification of new therapeutic opportunities and the acceleration of drug development. Ultimately, drug repurposing involves finding new therapeutic uses for existing drugs, despite the limited success obtained so far (9). While all three approaches can contribute to the development of new medications, their strategies and starting points differ (7).

## 2 Beyond the reductionist approach of a drug’s modes of action

In the context of mechanisms of action of drugs, the reductionistic approach involves studying the drug’s effects at the molecular, cellular, and physiological levels in order to elucidate how it functions within the body (8, 10). This approach focuses on identifying and understanding the specific molecular targets that drugs interact with, and the subsequent biochemical and physiological changes that occur as a result of drug-target interactions. At the molecular level, researchers investigate how drugs bind to specific proteins, receptors, enzymes, or other molecules involved in biological processes. They analyze the structure–activity relationship to determine how the drug’s chemical structure influences its interaction with the target, and how this interaction leads to molecular changes. Once the specific target or targets are identified, the reductionistic approach is applied at the cellular level. Researchers examine how the drug affects cellular signaling pathways, gene expression, protein synthesis, or other cellular processes. Understanding these cellular-level interactions helps elucidate how drugs modulate specific cell functions and influence overall physiological responses. At the physiological level, researchers investigate the effects of drugs on organ systems, whole organisms, and clinical outcomes. This includes studying how drugs affect organ function, systemic processes, and the overall disease state. By examining the drug’s impact on the entire organism, researchers gain insights into the broader therapeutic effects and potential side effects of the drug. Therefore, the reductionistic approach in mechanisms of action of drugs involves studying drugs at different levels of complexity, from the molecular to the physiological, to understand how they interact with biological systems. Yet, it almost exclusively focuses on modulating specific molecular targets of interest, namely, the qualitative and quantitative description of the drug/receptor interaction (11). As a matter of fact, currently, target-based drug discovery heavily dominates drug discovery approaches in both academia and the pharmaceutical industry. Little emphasis is placed on realistic disease conditions whereby the local tissue microenvironment and/or specific environmental factors might flexibly modulate a patient’s response. Indeed, due to the complexity of multifactorial diseases, drug intervention based on single-target drugs with high affinity, high selectivity, and strong potency may not fit well and does not always exhibit satisfactory efficacy with the network-based, inter-balanced regulation mode of the smart biological system (12, 13). Many “target-based” drugs have indeed numerous “off-target” therapeutic mechanisms (14). This has been a major bottleneck in the translation of potent single-target candidates, which inherently possess excellent potential but fail to demonstrate significant clinical impact due to disease mechanisms, which are in fact complex subnetworks within the interactome (15).

## 3 From systems biology to network pharmacology

Systems biology is an interdisciplinary field that combines biology, mathematics, and computer science to gain a holistic understanding of complex biological systems. It focuses on studying the behavior and interactions of various components within a biological system such as genes, proteins, cells, and organisms, with the aim of developing models and simulations to predict the behavior and responses of these systems. By integrating large-scale experimental data, computational modeling, and analysis techniques, systems biology seeks to unravel the underlying mechanisms and principles governing biological processes. It aims to provide insights into how these systems function, adapt, and respond to internal or external perturbations, such as diseases, drugs, or environmental factors. Systems biology has applications in various fields such as medicine, drug development, environmental science, and biotechnology. It can help to identify potential drug targets, optimize metabolic pathways for bioengineering, understand disease mechanisms, and develop personalized medicine strategies (16).

Systems biology has direct applications and strong connections to pharmacology (17, 18). By studying biological systems as a whole, systems biology can provide insights into how drugs interact with these systems, how they affect various components within the system, and how the system—as a whole—responds to the drug. Pharmacology traditionally focuses on studying the effects of drugs on specific targets or pathways within the body. However, systems biology takes a more holistic approach by considering the interactions and dynamic changes that occur across multiple biological components and their networks. With the help of systems biology, pharmacologists can gain a deeper understanding of the mechanisms of drug action, identify potential off-target effects or adverse reactions, predict drug efficacy in individual patients, and guide the development of personalized medicine strategies. Systems biology techniques, such as computational modeling and simulation, can help pharmacologists to analyze and interpret large datasets, integrate complex drug-target interactions, and predict the effects of drugs within the context of the entire biological system. By combining the principles and methods of systems biology with pharmacology, researchers can improve drug discovery processes, optimize drug development pipelines, and enhance the overall understanding of drug action and drug response in complex biological systems (19, 20).

A new discipline called network pharmacology (NP) has emerged which attempts to understand drug actions and interactions with multiple targets (21). Hopkins proposed NP as the next paradigm in drug discovery. This distinctive new approach to drug discovery can enable the paradigm shift from highly specific magic bullet-based drug discovery to multitargeted drug discovery. It attempts to discover new drug leads and targets and to repurpose existing drug molecules for different therapeutic conditions by allowing an unbiased investigation of potential target spaces (22). NP has the potential to provide new treatments to multigenic complex diseases and can lead to the development of e-therapeutics where the ligand formulation can be customized for each complex indication under every disease type. This can be expanded in the future and lead to customized and personalized therapeutics. Hopkins had suggested three strategies to the designers of multitarget therapies: the first was to prescribe multiple individual medications as a multidrug combination cocktail. Patient compliance and the danger of drug–drug interactions would be the expected drawbacks of this method. The second proposition was the development of multicomponent drug formulations. The change in metabolism, bioavailability, and pharmacokinetics of formulation as well as safety would be the major concerns of this approach. The third strategy was to design a single compound with selective polypharmacology. According to Hopkins, the third method is advantageous, as it would ease the dosing studies. Also, the regulatory barriers for the single compound are fewer compared to a formulation. An excellent example of this is metformin, the first-line drug for type II diabetes that has been found to have cancer-inhibiting properties (23). Integration of network biology and polypharmacology can tackle two major sources of attrition in drug development such as efficacy and toxicity. Also, this integration holds the promise of expanding the current opportunity space for druggable targets (24). However, these efforts require some guidance for selecting the right type of targets and new scaffolds of drug molecules. Traditional knowledge can play a vital role in this process of formulation discovery and repurposing existing drugs. By combining advances in systems biology and network pharmacology, it might be possible to rationally design the next generation of promiscuous drugs (25–27). Ultimately, advances in systems biology and high-throughput in-depth genomic profiling technologies along with an analysis of the successful and failed drugs uncovered that the prominent factor to determine drug sensitivity is the intrinsic robustness of the response of biological systems in the face of perturbations. In this regard, pleiotropic natural products are one of the promising strategies due to their multi-targeting and to lower side effects (28).

The following sections explore the experiences of our group as well as of other researchers with the thymic peptide hormone, thymosin alpha-1 (Tα1), as a truly phenotypic drug with pleiotropic activity.

## 4 Can thymosin alpha-1 be considered as one instance of phenotypic drug discovery?

Thymosin alpha-1 (Tα1, generic drug name: thymalfasin; trade name, Zadaxin) is a 28 amino-acid bioactive peptide originally isolated from the thymus (29–31). While Tα1 has been shown to have immunomodulatory effects and potential clinical applications, its discovery and development were not primarily driven by the screening of compounds in phenotypic assays but have been more target-driven, one example being offered by its unexpected activity in experimental cystic fibrosis (CF), which does represent an instance of PDD approach. Indeed, Tα1’s potential therapeutic applications were explored based on a variety of roles in different experimental setting, and not only in immune regulation and its interactions with specific immune cells and pathways (32, 33). Overall, Tα1 exhibits multiple actions across various biological systems and processes, making it a pleiotropic drug having multiple effects or actions on different biological systems. When it comes to mode of action, Tα1 operates through various mechanisms (Table 1). Relevant in this regard, the traditional interpretation of a drug that operates through multiple mechanisms of action is that it has the potential to produce a broader and potentially more effective therapeutic effect compared to a drug that acts through a single mechanism. This is based on the idea that targeting multiple biological pathways related to a disease or condition can lead to a more comprehensive and possibly synergistic treatment approach. However, it’s important to note that the traditional interpretation does not guarantee that a drug with multiple mechanisms of action will always be more effective or have fewer side effects than a drug with a single mechanism of action, which could represent one major drawback of PDD. The actual therapeutic outcomes and safety profile of any drug depend on its specific properties, the nature of the disease being treated, and individual patient characteristics. As illustrated above—and in line with the principles of NP, as derived by systems biology—an emerging concept is likewise that such drugs may have a more comprehensive or versatile effect on the body, potentially affecting various pathways or targets to produce their overall therapeutic outcome. This multidimensional approach can be advantageous in addressing complex conditions or symptoms that may have diverse underlying causes. In other terms, they might provide broader therapeutic benefits: Drugs with multiple mechanisms of action may have the potential to address a wider range of symptoms or disease processes, potentially offering a more holistic approach to treatment.

**Table 1:** Pleiotropic mode of action of thymosin alpha-1.

Mode of action	*K_D_*	*In vitro* functional activity	*In vivo* functional activity
Binding to the N- and C-domains of the angiotensin-converting enzyme resulting in inhibition of its expression in human lung epithelial cells (34, 35)	17.4 nM (35)	2.5 μg/mL (35)	NA
Activation of the MAPK/JNK (36), PI3K/Akt/mTOR (37), canonical and non-canonical NF-κB kinases and IRFs signaling pathways for immunomodulation and immunostimulation (38, 39)	No *K_D_* available	10–100 ng/mL range (36), 130 μM (37), 100 ng/mL (38)	NA
Regulation pf transcription and/or DNA replication for antigen expression (40)	No *K_D_* available	1 μg/mL	NA
Downregulation of STAT3 phosphorylation and transcription of MMP2 in PD-L1 high-expressing NSCLC cells to suppress migration and invasion (41)	No *K_D_* available	90–180 μM	20 μg/g
Binding to the VIP receptors in different types of cells with a low affinity (42)	No *K_D_* available	1,600 nM	NA
Binding inhibitory activity of galectin-1 for immune homeostasis (43)	*K_D_* = 46 μM	50–200 μg/mL range	NA
Interaction with phosphatidylserine via its N terminus insertion to promote nearby proteins and/or receptors activation and biological signaling (44)	No *K_D_* available	4–8 mM	NA
Binding to serum albumin (45) and to hyaluronan (46) through the lysine residues of the sequence LKEKK at the C terminal domain, to regulate functional activity	*K_D_* = 0.4 mM (45) *K_D_* = 2.2 mM (46)	NA	NA
Toll-like Receptor (TLR)2, TLR4 and TLR9 activation to finely regulate immunity and tolerance in infection and antitumor immunity (47–49)	No *K_D_* available	20 ng/mL (47)	400 μg/kg (48) 200 μg/kg (49)
Inhibition of ROS production and increase of catalase, peroxide dismutase and glutathione peroxidase in human cells (50, 51)	No *K_D_* available	3–48 μg/mL (50)	NA
Activation of the enzyme indoleamine 2, 3-dioxygenase (IDO)-1 to promote autophagy, restrain inflammation and provide immune tolerance (52, 53)	No *K_D_* available	100 ng/mL (52)	200 μg/kg (52)
Activation of the Aryl hydrocarbon Receptor (AhR)/IL-22 axis to protect mice from the metabolic disorders associated with high caloric intake (54)	No *K_D_* available	NA	20–200 μg/kg
Chaperon activity in experimental cystic fibrosis to facilitate the correct folding and trafficking of the mutant CFTR protein, thereby improving their function (52, 53)	No *K_D_* available	100 ng/mL (52)	200 μg/kg (52)

*K_D_*, dissociation constant. Shown are ranges of concentrations used *in vitro* or doses *in vivo*. NA, not available.

The following is an overview of the pleiotropic role of Tα1 in both physiology and medicine.

## 5 Tα1 in physiology

**Figure 1:**
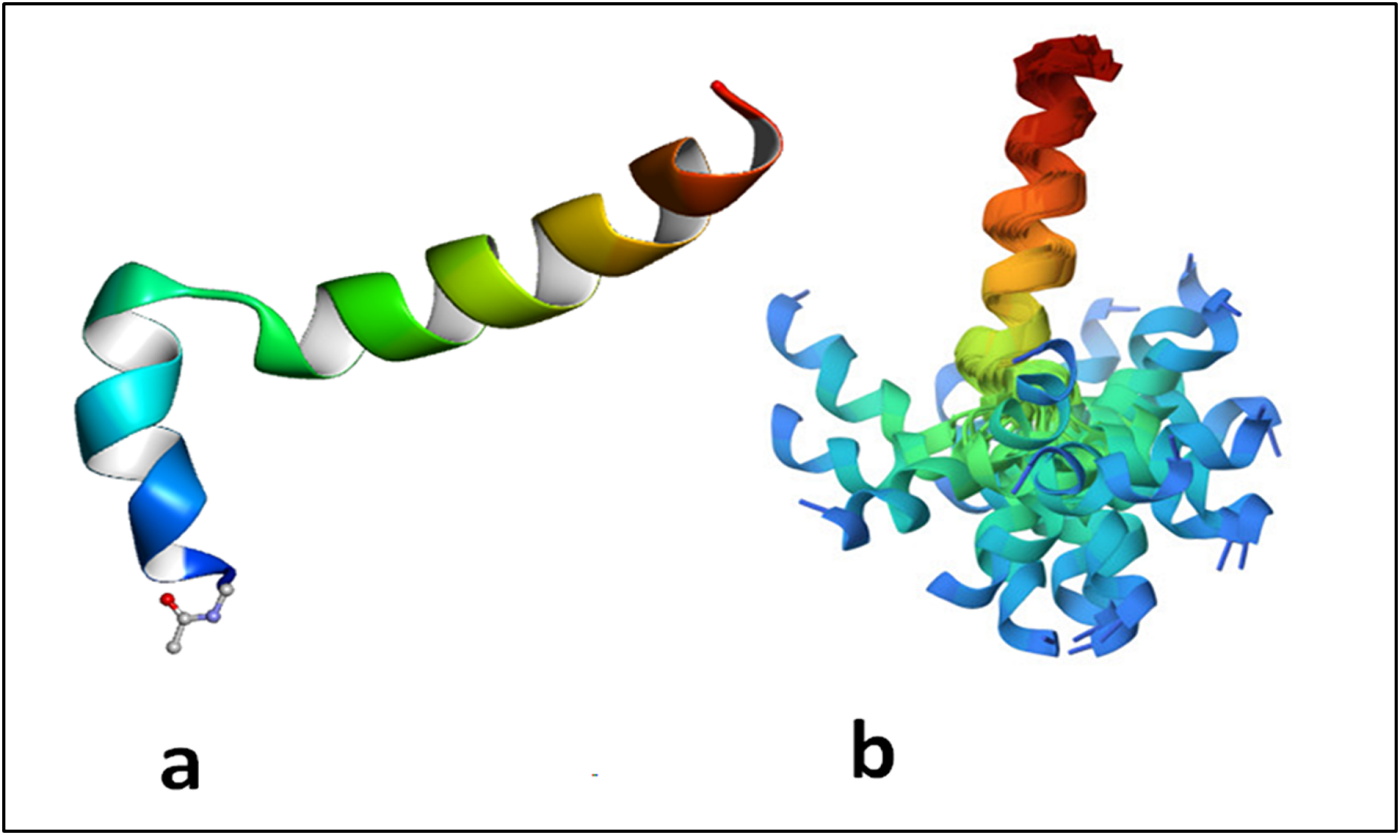
Thymosin alpha 1 is an intrinsically disordered protein which assumes a two helical conformation with a central unstructured loop. The structure in phosphatidylserine membrane is reported in **(A)** where it evident the role of the acetyl group linked to Ser 1 inserted in the membrane. In **(B)** the structures obtained by the constraints from NMR spectroscopy maintained aligned the C terminal domains of the protein. The N terminal domain appears spread due to disorder of the mobile central loop. Adapted from PDB 2MNQ.

Tα1 is highly acidic peptide produced by asparagine endopeptidase cleavage of prothymosin α in numerous mammalian organs, including the thymus, spleen, lung, kidney, brain, and blood, with the largest concentration in the thymus (55). Under natural conditions, Tα1 is a short, acidic and highly charged, and inherently disordered protein but in the presence of cellular membranes or under low pH conditions, it assumes a partly structured conformation through interaction with other naturally existing proteins (46, 56, 57). Thus, despite the lack of specific receptors underlying the activity of Tα1, upon folding on a membrane with negative charge by exposing phosphatidylserine on the surface, Tα1 blocked by its acetylated N-terminal on the membrane may interact with receptors on or near the membrane resulting in a cascade of signaling responses of biological meaning (58, 59). Under many respects, Tα1 can be considered an example of a protein with an intrinsically disordered domain or region (IDRs), similar to its precursor prothymosin alpha (60) (Figure 1). As mentioned above, owing to the absence of a fixed 3D structure, IDRs are—in general—more sensitive to their physicochemical surroundings than folded domains. This sensitivity means IDRs are well suited to act as intracellular sensors, whereby sequence-encoded conformational biases are altered by varying salt, pH, temperature, metabolites, and other solution changes (61, 62). Of interest, IDRs can also contribute to the formation of biomolecular condensates through intracellular phase transitions (63), thus functioning to concentrate proteins and nucleic acids involved in diverse processes, including RNA metabolism, ribosome biogenesis, the DNA damage response and signal transduction. Thus, IDRs, including Tα1 (57), are excellently positioned to serve as discerning detectors and effectors of cellular physicochemistry and their disordered nature appears to be the key for understanding their promiscuous binding behavior with different targets.

Tα1 is, indeed, endowed with a plethora of immunoregulatory activities (39, 64, 65), which include: (1) Maturation, activation and prevention of apoptosis of various immune cells, such as T and B lymphocytes and Natural Killer cells, via different signaling pathways, including TLRs (36–40, 47–49); (2) Activation of CD8^+^ T cells for cross-priming in antitumor and antiviral responses, via transcriptional regulation of MHC class I expression (40) and immunostimulation (66, 67); (3) Activation of innate immune cells for antimicrobial activity (68); (4) Activation of antigen presenting function of different dendritic cells subsets via selective TLR stimulation for immunity and tolerance in infection and antitumor immunity (47–49, 52, 53); (5) Regulation of cytokines for increased production of interferon-γ (IFN-γ), IFN-α, interleukin-2 (IL-2), IL-6, and IL-10 and decreased production of inflammatory IL-1β and TNF-α via different receptor signaling pathways (37–39, 47–49, 69, 70); (6) Induction of immune tolerance via the activation of the tolerogenic IDO1 pathway (52, 53); (7) Metabolic activity via the AhR/IL-22 axis and the control of lipid peroxidation in experimental metabolic disorders (54, 71); (8) Metabolic regulation of the oxidative/anti-oxidative stress pathways in preclinical murine and human preclinical settings (50, 51); (9) Direct antitumor activity via PTEN-mediated apoptosis (37) and other mechanisms (67), including suppression of migration via inhibition of STAT3-MMP2 signaling (41). Overall, it appears that Tα1 is capable of a multifaceted, pleiotropic immune activation, resulting in apparently opposing effects on the immune system, an activity that points to its context-dependent activity, as long suggested (39). Indeed, the exploitation of the intersection between canonical and noncanonical signaling pathways of NF-κB, a family of transcription factors that play a central role in the stress response and inflammation, and of different Interferon Regulatory Factors is likely underlying its pleiotropic activity. Although primarily exerting a fine immunoregulatory activity, Tα1 also modulated central nervous system activity (72, 73) by poorly defined molecular mechanisms.

## 6 Potency and pharmacokinetics implications

It is clear that much needs be done for a full understanding of both the mechanism(s) of action and the dose required for the effects of Tα1 in both physiology and medicine. Apparently, very few studies have described the binding of Tα1 to putative targets and the relative dissociation constant (Table 1), an observation to which its IDR behavior affecting various pathways or targets likely contributes. As a matter of fact, as cited by Sargent and Schweitzer: “a direct ligand-receptor reaction is replaced by multiple sequential steps including surface accumulation of charged ligands, ligand-membrane interactions, and ultimately binding to the receptor itself” in the case of peptides interacting with their targets (74). Meaning that the apparently measured dissociation constant is a function of the whole system rather than just the receptor. Accordingly, the concentrations of Tα1 also vary depending on the type of study and biological system being used. For instance, low (typically range from 1 to 10 ng/mL), moderate (from 10 to 100 ng/mL) and high—up to 1,000 ng/mL—concentrations have been reported in many *in vitro* cellular studies. Drugs used within this range typically indicate a moderate to high interaction with their receptor, if any. Similarly, *in vivo*, doses of Tα1 can vary greatly depending on the animal model, route of administration, and type of pathological condition being treated, ranging from 0.1 to 1 mg/kg body weight, administered subcutaneously or intraperitoneally to mice and rats, and from 1 to 3 mg administered subcutaneously or intramuscularly to humans. Consistent with the *in vitro* studies, drugs used *in vivo* in a range from 0.05 to 1 mg/kg typically indicate a moderate to high interaction with host’s cells. This concentration range suggests a significant pharmacological effect, implying that the drug is active at relatively low doses. There are no reported instances of deliberate or accidental overdosage in humans. Animal toxicology studies have shown no adverse reactions in single doses up to 20 mg/kg and in repeated doses up to 6 mg/kg/day for 13 weeks, which were the highest doses studied. The highest single dose tested in animals represents 800-times the clinical dose.

In addition, as to the pharmacokinetics of Tα1 in humans, the available information is so far limited. Single-dose and multiple-dose pharmacokinetic studies were conducted in healthy volunteers. Doses ranging from 0.8 to 6.4 mg were evaluated in single-dose studies and daily doses of 1.6 and 3.2 mg were evaluated in multiple-dose studies of 5 or 7 days’ duration. Tα1 is rapidly absorbed with a T_max_ (Time to peak drug concentration) of approximately 2 h. A dose-proportional increase was present in serum levels for C_max_ and AUC. The C_max_ values were 39, 63, 85, and 130 ng/mL and the AUC values were 124, 261, 314, and 679 ng/h/ml, respectively, at 0.8, 1.6, 3.2, and 6.4 mg. Serum levels returned to basal levels by 24 h after administration. The serum half-life was approximately 2 h, and there was no evidence of accumulation following multiple administrations. The urine excretion accounted for up to approximately 50–60% of the single dose and 24% following multiple doses. A multiple-dose study in healthy volunteers (at doses of 1.6, 8, and 16 mg twice weekly for 4 weeks) revealed doses to be well tolerated. A preliminary evaluation of serum drug levels of Tα1 indicated a dose-proportional increase; the approximate C_max_ levels were 30, 180, and 310 ng/mL at doses of 1.6, 8, and 16 mg, respectively. Peak levels occurred at 1 to 2 h, and there was no evidence of accumulation. In a pharmacokinetics study in lung cancer, the subjects in the loading dose treatment arm showed similar results as above. Plasma levels returned to basal levels within 24 h of administration and there was no evidence of accumulation (75, 76).

## 7 Tα1 in medicine

Consistent with the reduced serum levels of Tα1 in chronic inflammatory autoimmune diseases [below 1 ng/mL in the bloodstream as compared to 28.74 (17.98–70.25 interquartile range) versus 78.96 (40.80–130.13) in healthy females and males, respectively] (77), Tα1 has been extensively studied for its potential therapeutic applications in various medical conditions, with an excellent safety profile (78), including:

### 7.1 Infectious diseases

Tα1 has been investigated as an adjuvant therapy for viral, bacterial, and fungal infections (33, 66, 79, 80). It has been used to treat a variety of illnesses, including chronic hepatitis B and C, (81–83), acquired immune deficiency syndrome (84), bacterial and mold pneumonia (33), sepsis (85, 86), and, most recently, COVID-19 (80, 87–103). However, alongside beneficial effects (87, 92–97), clearly supporting the use of Tα1 in COVID-19 via multiple immunity-enhancing and anti-inflammatory protective mechanisms (95), no effects (98–101) or poor clinical outcomes (102) were also observed in COVID-19 patients treated with Tα1, these being inconsistent findings to which the heterogeneity of the disease, including the gender (87, 98, 103), could contribute. Interestingly, Tα1 showed beneficial effects either in the acute COVID-19 phase or in reinfection even in elderly patients (89), thus pointing to a potential immunorestorative effect of Tα1 in aging, as suggested (104). Its synthetic derivative, thymalfasin, has been integrated into various clinical products and is now approved in over 35 countries for the treatment of hepatitis B and C.

### 7.2 Cancer

It is being explored as an immunotherapeutic agent for certain types of cancers, such as melanoma, hepatocellular carcinoma, and lung cancer (105), either alone or combined with chemotherapy (106, 107) or radiotherapy or used as postoperative adjuvant therapy (108). In combination with Dacarbazine, Tα1 showed a 3-fold increase in response rate in patients with stage IV melanoma compared to Dacarbazine alone (109). The antitumor activity of Tα1 occurs through different pathways, including inhibition of cell proliferation and induction of apoptosis (37), promotion of immunosurveillance by increasing the expression of MHC I (40) and tumor antigens (110), counteraction of the immunosuppressive effects associated with conventional chemotherapy, radiotherapy and targeted therapy (51, 67, 111, 112). More recently, Tα1 was found to reverse M2 polarization of efferocytosis-activated macrophages in cancer patients (113) and, in the context of immune check-point inhibitor therapy, to enhance anti-tumor activity (114) via the promotion of dendritic cell activity (115).

### 7.3 Autoimmune diseases

Tα1 shows potential in the management of autoimmune diseases like rheumatoid arthritis, multiple sclerosis, and systemic lupus erythematosus, likely through its anti-inflammatory activity (116, 117).

### 7.4 Immunodeficiency disorders

Tα1 has been shown to boost immune function in individuals with primary immunodeficiency disorders or those who are immunocompromised (118). The National Health Commission of China included Tα1 as an alternative treatment option for COVID-19 patients with lymphocytopenia or immunodeficiency.

### 7.5 Other disorders

Tα1 showed remarkable effects in the treatment of acute respiratory distress syndrome, severe acute respiratory syndrome and acute exacerbation of chronic obstructive pulmonary disease, gastrointestinal and systemic infectious disorders (70, 90, 119, 120). In cystic fibrosis (CF), in which the hyperinflammatory state is associated with early and nonresolving activation of innate immunity, which impairs microbial clearance and promotes a self-sustaining condition of progressive lung damage, Tα1significantly alleviated the symptoms associated with the hyperinflammatory pathology. A finding consistent with its ability to activate the tolerogenic IDO1/Treg pathway (52–54). Unexpectedly, however, Tα1 also improved the cellular trafficking of CFTR via autophagy, a finding consistent with the ability of IDO1 to activate autophagy. Thus, by providing a multipronged attack against CF, i.e., restraining inflammation and correcting the basic defect, Tα1 favorably opposed CF symptomatology in preclinical relevant disease settings. This could represent a teaching example of how the inherent complexity of the pathogenic mechanisms requires a polyfunctional drug and emphasizes the powerful tool of PDD.

## 8 Conclusion

It is clear that Tα1 is a definite example of PDD, because its development was based on observed effects on the immune system rather than a specific, known molecular target. Originally identified as a thymic hormone with immune-modulating properties, Tα1 was later found to have potential therapeutic applications in enhancing immune function, combating various diseases and regulating cellular proteostasis. Tα1 is currently exploited therapeutically because and/or despite its pleiotropic activity. Thymalfasin (Zadaxin from SciClone, the only thymosin-based FDA-approved drug) is indeed used in over 35 countries for the treatment of hepatitis B and C, melanoma and a variety of illnesses, including acquired immune deficiency syndrome, respiratory infections, SARS-CoV-2 and sepsis. However, a number of patents on Tα1 and other thymosins have recently been reported in disparate clinical settings, implying that the therapeutic utility of these peptides will be further expanded. Thus, its development as a therapeutic agent exemplifies the successful use of a phenotypic approach to drug discovery, where the observed effects on a specific biological system led to its development as a potential treatment for various conditions. Not secondarily, Tα1 belongs to NP due to its ability to modulate complex biological networks involved in important biological processes. Additionally, NP approaches can be used to identify potential drug targets within the immune network that may be modulated by Tα1, providing insights into its mode of action and potential for combination therapies. Its influence extends across multiple levels of biological organization, including gene expression, protein–protein interactions, and cellular signaling pathways. By affecting these interconnected biological networks, secondarily, Tα1 can have broad-ranging effects on biological functions in health and diseases. While the inherent poor *in vivo* stability of peptides (121) is being overcome by the development of long-acting Tα1 by chemical modification (122, 123) or microbial engineering (124, 125), much remains to be learned on how Tα1 can be accommodated within the multidimensional nature of human diseases and its pharmacology. Ultimately, the dependency of a drug’s pharmacological activity on the pathological context is an emerging concept in pharmacology, exemplified by the context-dependent pharmacological effects of metformin on the immune system whereby metformin exhibits immunostimulatory effects in tumor immunity but has immunosuppressive effects in the context of autoimmune or inflammatory diseases (126). Moreover, a context-dependent signaling characterizes the nuanced behavior of G protein-coupled receptors in physiologically relevant contexts (127). A similar context–dependent activity is likely characterizing the effects of Tα1 on the immune system being experimental and clinical evidence highlighting either its immune-enhancing effects on anti-tumor immunity or its tolerogenic, anti-inflammatory potential in inflammatory conditions. Certainly, a little peptide has taught us that the “single compound, single target” drug development model has inherent limitations and that various networks constructions are needed to decipher its pleiotropy. Overall, Tα1’s impact on interconnected biological networks, particularly within the immune system, aligns with the principles of NP and underscores its relevance within this field. Ultimately, classic views of drug action should be questioned in the light of the presence of disordered domains—almost 70% of protein domains might be disordered—that confer a degree of “fuzziness and imprecision” as an essential feature of protein interactions. As recently highlighted, “being disordered makes proteins versatile communicators, able to respond rapidly to changes in the cell, binding to different partners and transmitting different signals depending on the circumstance” (128).

## Author contributions

EG: Conceptualization, Writing – review & editing. MP: Writing – review & editing. CM: Writing – review & editing. CC: Writing – review & editing. PP: Writing – original draft, Writing – review & editing. LR: Conceptualization, Funding acquisition, Writing – original draft, Writing – review & editing.
